# Tibia shaft fractures: costly burden of nonunions

**DOI:** 10.1186/1471-2474-14-42

**Published:** 2013-01-26

**Authors:** Evgeniya Antonova, T Kim Le, Russel Burge, John Mershon

**Affiliations:** 1 Eli Lilly and Company, Indianapolis, USA

**Keywords:** Tibia shaft fractures, Nonunions, Healthcare resource utilization, Costs, Opioids

## Abstract

**Background:**

Tibia shaft fractures (TSF) are common for men and women and cause substantial morbidity, healthcare use, and costs. The impact of nonunions on healthcare use and costs is poorly described. Our goal was to investigate patient characteristics and healthcare use and costs associated with TSF in patients with and without nonunion.

**Methods:**

We retrospectively analyzed medical claims in large U.S. managed care claims databases (Thomson Reuters MarketScan®, 16 million lives). We studied patients ≥ 18 years old with a TSF diagnosis (ICD-9 codes: 823.20, 823.22, 823.30, 823.32) in 2006 with continuous pharmaceutical and medical benefit enrollment 1 year prior and 2 years post-fracture. Nonunion was defined by ICD-9 code 733.82 (after the TSF date).

**Results:**

Among the 853 patients with TSF, 99 (12%) had nonunion. Patients with nonunion had more comorbidities (30 vs. 21, pre-fracture) and were more likely to have their TSF open (87% vs. 70%) than those without nonunion. Patients with nonunion were more likely to have additional fractures during the 2-year follow-up (of lower limb [88.9% vs. 69.5%, P < 0.001], spine or trunk [16.2% vs. 7.2%, P = 0.002], and skull [5.1% vs. 1.3%, P = 0.008]) than those without nonunion. Nonunion patients were more likely to use various types of surgical care, inpatient care (tibia and non-tibia related: 65% vs. 40%, P < 0.001) and outpatient physical therapy (tibia-related: 60% vs. 42%, P < 0.001) than those without nonunion. All categories of care (except emergency room costs) were more expensive in nonunion patients than in those without nonunion: median total care cost $25,556 vs. $11,686, P < 0.001. Nonunion patients were much more likely to be prescribed pain medications (99% vs. 92%, P = 0.009), especially strong opioids (90% vs. 76.4%, P = 0.002) and had longer length of opioid therapy (5.4 months vs. 2.8 months, P < 0.001) than patients without nonunion. Tibia fracture patterns in men differed from those in women.

**Conclusions:**

Nonunions in TSF’s are associated with substantial healthcare resource use, common use of strong opioids, and high per-patient costs. Open fractures are associated with higher likelihood of nonunion than closed ones. Effective screening of nonunion risk may decrease this morbidity and subsequent healthcare resource use and costs.

## Background

Tibia shaft fractures are common but unanticipated trauma in adults resulting in painful and prolonged recovery, often associated with complications. The U.S. National Center for Health Statistics reported annual incidence of 492,000 fractures of tibia, fibula, and ankle [1]. Tibia and fibula fractures annually result in 77,000 hospitalizations accounting for 569,000 hospital days and 825,000 physician office visits [2]. The U.S. Agency for Healthcare Research and Quality (AHRQ) reported 151,966 hospital discharges for which tibia/fibula fracture diagnosis was a reason for a principal procedure in 2007 (Healthcare Cost and Use Project, AHRQ) [3]. A high proportion of Medicare patients – adults aged 65 or older – with tibia fractures undergo an acute inpatient stay (70%), post-acute inpatient stay (50%), and home health care (38%) as well as outpatient visits and physical and occupational therapy [4]; such estimates are missing for young and middle-age adults who also frequently get tibia fractures [5,6]. Tibia fractures are treated medically, and healthcare use depends on treatment options, which, in turn, vary by injury type and severity and the presence of complications [5,7].

Fracture nonunion (sometimes referred to as “delayed union”) is a common complication of a tibia fracture; it indicates that fracture healing is not happening in a timely fashion [5,8]. Nonunions put additional burden on the patient because they prolong the disability and are associated with substantial pain [9,10]. There is no standard definition of nonunion, and some authors have defined tibia nonunion as a fracture that has not united without additional surgical or nonsurgical intervention within 6–9 months [8], whereas others waited for six-month to perform surgeries to correct nonunions [11]. 

A common approach to delayed unions is expectant management, accompanied by non-invasive therapies such as low-intensity pulsed ultrasound [12-14], or vibration [12]. When healing fails within a clinically reasonable time period (6–9 months), a second surgical intervention, aiming to stabilize the fracture, is inevitable [8,11]. Additional therapies used during the surgery, such as bone morphogenetic proteins (BMP’s), may further help bone healing, but they are costly.

Nonunions naturally require more healthcare services than fractures without non-unions because of the repeated surgical intervention and the extended patient pain and disability. Understanding of patient characteristics, healthcare use, and costs associated with tibia fracture nonunion is critical to understanding the clinical and economic burden.

Although previous studies investigated nonunion-related healthcare use and costs [15-18], they had a number of limitations: small sample size (n < =27) [15-18], no comparison to patients without nonunion [15-18], outdated estimates [15,18], single healthcare setting [15], and focus on limited therapies: pulsed low-intensity ultrasound [18], autologous-iliac-crest-bone-graft, or bone morphogenetic protein-7 (BMP-7) [16,17]. These shortcomings limit the reliability of the estimates, external validity (the ability to generalize to other patient populations and healthcare settings), and the ability compare with costs of fractures with nonunion to those without nonunion. To address these shortcomings, we conducted an analysis of large U.S. medical claims databases that reflects multiple healthcare settings and therapies. The aim of our study was to describe patient characteristics, healthcare resource use, and costs associated with tibia shaft fractures overall and by nonunion status.

## Methods

### Data

We used data from the Thomson Reuters Marketscan® Research Databases. These retrospective claims databases are fully compliant with the Health Insurance Portability and Accountability Act (HIPAA). The data capture person-specific healthcare use, expenditures, and enrollment in inpatient, outpatient, prescription-drug, and carve-out services^a^ from a selection of large employers, health plans, and government and public organizations. The databases link paid claims and healthcare encounter data to detailed patient information (for multiple sites and types of providers) over time. The databases include private sector health data from approximately 100 payers and more than 500 million claim records throughout the United States. The examined data spanned the years 2005 through 2008 and reflected approximately 16 million patient lives. This study did not enroll human subjects.

### Patient sample

This study included adult patients diagnosed with a tibia shaft fracture (sometimes accompanied with a fibula fracture), which included ICD-9-CM diagnostic codes 823.20, 823.22, 823.30, and 823.32. The fractures occurred during the calendar year 2006 (the index period). Patients were required to have continuous insurance coverage in the year prior to the index period (2005) and during the two years following the index period (2007–2008). Please see Online Additional file 1 for more details. We excluded patients who were diagnosed with a tibia fracture in the calendar year prior to the index period and patients who were younger than 18 years at the time of their tibia shaft fracture. Nonunion was defined by ICD-9 code 733.82 recorded after the initial tibia fracture date.

### Measures

We extracted the following data for each patient in the sample: age, sex, comorbidities, additional fractures, non-union status (yes or no), open vs. closed status of the tibia fracture, use of pain medications, healthcare resource use, and medical costs. Healthcare use and costs included all inpatient, outpatient, and pharmacy services billed for during the 24 months after the tibia fracture. Costs were divided into tibia related (whenever tibia fracture diagnosis code was assigned to the medical claim) and non-tibia related (whenever tibia fracture diagnosis code was not assigned to the medical claim). Opioids were classified as “weak” (tramadol and hydrochloride) and “strong” (all others). We assigned “open” fracture status to the cases with an open fracture record in the tibia shaft related claims. Cases with fractures (other than the index fracture) recorded in claims in the 2-year follow-up period received the “additional fracture” status.

### Analyses

We described demographic characteristics (age and sex) for all tibia fracture patients (total and by nonunion status). We compared healthcare resource use and medical costs of tibia shaft fracture in patients with and without nonunion. The Charlson comorbidity index (CCI) [18] was used to estimate patient general health status. Costs and health care resources were annualized and converted into 2006 U.S. dollars. Costs were analyzed as tibia-related, non-tibia related, and total.

### Statistical methods

In statistical comparisons, we used Chi-square tests to examine differences in patient counts. We used a nonparametric technique - Wilcoxon test - to compare continuous variables (to minimize the influence of cost outliers). P values < 0.05 were considered to be statistically significant. We conducted analyses using SAS, version 9.3 (Cary, NC).

Internal Review Board (IRB) approval was not required for this retrospective database study because the data were de-identified and followed the HIPAA guidelines prior to our analyses.

## Results

A total of 853 tibia fracture patients were selected for the study based on the inclusion and exclusion criteria above.

### Tibia fracture patient characteristics: age and sex

**Figure 1:**
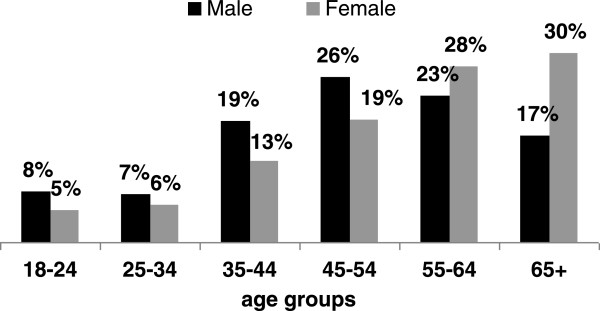
Distribution of tibia fractures in age categories by sex.

Average patient age at the time of fracture varied by sex. The male tibia fracture patients were most likely to be between their forties and fifties (Figure 1) with the mean age 50 years old (y.o.) (median age = 51 y.o.). In contrast, the female tibia fracture patients were most likely to be 65 y.o. or older, and the mean age at the time of the fracture in women was 56 y.o. (median age = 57 y.o.). Age difference between sexes was statistically significant (p < 0.001).

### Tibia fracture patient characteristics by nonunion status

Out of 853 patients with a tibia shaft fracture, 99 (12%) patients had a nonunion diagnosis associated with their tibia fracture treatment. Patients with nonunion had characteristics similar to those without nonunion: there was no statistically significant differences in age, sex, region of residence, type of insurance coverage, or CCI (Table 1).

**Table 1:** Patient characteristics by the nonunion status

**Characteristic**	**All N = 853**		**With nonunion N = 99**		**Without nonunion N = 754**		**P values**
	**N**	**%**	**N**	**%**	**N**	**%**	
Sex:							
Male	378	44.3%	47	47.5%	331	43.9%	0.50**†**
Female	475	55.7%	52	52.5%	423	56.1%	
Age at Index Date:							
Median	54		53		55		0.54**‡**
Mean	53		52		53		
SD	16		15		17		
Range	18–93		18–87		18–93		
Charlson Comorbidity Index:							
Median	1		1		1		0.45**‡**
Mean	2		2		2		
SD	2		2		2		
Range	0–15		0–7		0–15		
Additional fractures:							
Lower Limb	612	71.70%	88	88.90%	524	69.50%	< 0.001**†**
Upper Limb	90	10.60%	12	12.10%	78	10.30%	0.59**†**
Spine and Trunk	70	8.20%	16	16.20%	54	7.20%	0.0022**†**
Skull	15	1.80%	5	5.10%	10	1.30%	0.008**†**
Open fracture status							
Open	132	15.5%	30	30.3%	102	13.5%	< 0.001**ξ**
Closed	721	84.5%	69	69.7%	652	86.5%	

Notes: † P-value is based on the T-test, ‡ P-value is based on the Wilcoxon test; **ξ -** P-value is based on the Chi-Square test; SD – standard deviation.

Patients with open tibia shaft fractures were much more likely to develop a nonunion than those with closed fractures. Ten percent (69/721) of patients with a closed fracture developed a nonunion compared with 23% (30/132) of open fractures patients (p < 0.001, Table 1). Also, patients with nonunion were more likely to have additional fractures during the 2-year follow-up (of lower limb [88.9% vs. 69.5%, P < 0.001], spine or trunk [16.2% vs. 7.2%, P = 0.002], and skull [5.1% vs. 1.3%, P = 0.008]) than those without nonunion (Table 1).

### Healthcare resource use

Tibia fracture patients consumed a substantial amount of prescription medications (Table 2). Overall, more than nine out of ten (93.2%) tibia shaft fracture patients used prescription medications during the 2-year follow-up period. The most common prescription medications among all tibia shaft patients were strong opioids (78.0%) and non-steroidal anti-inflammatory drugs (NSAID, 35.3%).

**Table 2:** Prescription medications use in patients with a tibia shaft fracture by nonunion status

**Prescription Medications**	**All tibia fractures (N = 853)**	**With Nonunion (N = 99)**	**Without Nonunion (N = 754)**	**P values**
Any Pain Medication				
Patients on therapy, n (%)	795 (93.2%)	98 (99.0%)	697 (92.4%)	0.009**†**
· Average therapy duration, months (SD)	N/A	N/A	N/A	X
Strong Opioids				
· Patients on therapy, n (%)	665 (78.0%)	89 (89.9%)	576 (76.4%)	0.002**†**
· Average therapy duration, months (SD)	3.2 (5.5)	5.4 (6.8)	2.8 (5.2)	< 0.001**‡**
Prescription NSAIDs				
· Patients on therapy, n (%)	301 (35.3%)	47 (47.5%)	254 (33.7%)	0.007**†**
· Average therapy duration, months (SD)	3.6 (4.9)	4.3 (6.0)	3.5 (4.7)	0.67**‡**
Weak Opiods				
· Patients on therapy, n (%)	247 (29.0%)	34 (34.3%)	213 (28.2%)	0.21**†**
· Average therapy duration, months (SD)	2 (3.5)	2.2 (3.5)	2.0 (3.5)	0.13**‡**
Benzodiazepines				
· Patients on therapy, n (%)	235 (27.5%)	36 (36.4%)	199 (26.4%)	0.037**†**
· Average therapy duration, months (SD)	7.2 (7.6)	7.4 (8.5)	7.1 (7.4)	0.68**‡**
Antidepressants: SSRI				
· Patients on therapy, n (%)	220 (25.8%)	30 (30.3%)	190 (25.2%)	0.28**†**
· Average therapy duration, months (SD)	11.6 (7.2)	8.1 (6.6)	12.1 (7.1)	0.003**‡**
Corticosteroids				
· Patients on therapy, n (%)	201 (23.6%)	27 (27.3%)	174 (23.1%)	0.36**†**
· Average therapy duration, months (SD)	1.9 (4.1)	2.3 (4.4)	1.9 (4.1)	0.21**‡**
Non-Benzodiazepines Sedative/Hypnotics				
· Patients on therapy, n (%)	197 (23.1%)	29 (29.3%)	168 (22.3%)	0.12**†**
· Average therapy duration, months (SD)	4.4 (5.7)	3.8 (3.9)	4.5 (6)	
Muscle Relaxants				
· Patients on therapy, n (%)	174 (20.4%)	25 (25.3%)	149 (19.8%)	0.2**†**
· Average therapy duration, months (SD)	3.9 (6)	5.0 (6.5)	3.7 (6.0)	0.061**‡**
Inject Corticosteroid				
· Patients on therapy, n (%)	168 (19.7%)	28 (28.3%)	140 (18.6%)	0.022**†**
· Average therapy duration, months (SD)	0.1 (0.1)	0.1 (0.1)	0.1 (0.1)	0.75**‡**
Anticonvulsant/Antiepileptic				
· Patients on therapy, n (%)	128 (15.0%)	23 (23.2%)	105 (13.9%)	0.015**†**
· Average therapy duration, months (SD)	8.3 (7.1)	9.1 (7.6)	8.1 (7.0)	0.59**‡**
Antidepressants: Other				
· Patients on therapy, n (%)	92 (10.8%)	13 (13.1%)	79 (10.5%)	0.42**†**
· Average therapy duration, months (SD)	9.7 (8.0)	8.4 (7.7)	10.0 (8.1)	0.76**‡**
Antidepressants: SNRI				
· Patients on therapy, n (%)	77 (9.0%)	19 (19.2%)	58 (7.7%)	< 0.001**†**
· Average therapy duration, months (SD)	10 (7.3)	12.0 (7.8)	9.3 (7.0)	0.22**‡**
Antidepressants: TCAs				
· Patients on therapy, n (%)	71 (8.3%)	11 (11.1%)	60 (8.0%)	0.29**†**
· Average therapy duration, months (SD)	8.5 (7.7)	8.4 (9.1)	8.5 (7.5)	0.6**‡**
COX-2 Inhibitors				
· Patients on therapy, n (%)	62 (7.3%)	10 (10.1%)	52 (6.9%)	0.25**†**
· Average therapy duration, months (SD)	7.2 (7.2)	6.7 (8.0)	7.3 (7.2)	0.6**‡**
Analgesics/Antipyretics, NEC				
· Patients on therapy, n (%)	13 (1.5%)	1 (1.0%)	12 (1.6%)	1.0**†**
· Average therapy duration, months (SD)	1.3 (2.7)	2 (MISS)	1.3 (2.8)	0.28**‡**

Notes: † P-value is based on the *T*-test, ‡ P-value is based on the Wilcoxon test; SD – standard deviation; NSAID - non-steroidal anti-inflammatory drugs; SSRI - selective serotonin reuptake inhibitors; SNRI - Serotonin–norepinephrine reuptake inhibitors; TCA - Tricyclic antidepressants; NEC – not elsewhere classified.

A higher proportion of nonunion patients used medications than those without nonunion (99.0% vs. 92.4%, P = 0.009). This disparity was driven by the following medications: strong opioids (89.9% vs. 76.4%, P = 0.002), prescription NSAID’s (47.5% vs. 33.7%, P = 0.007), benzodiazepines (36.4% vs. 26.4%, P = 0.037), injectable corticosteroid (28.3% vs. 18.6%, P = 0.022). Nonunion patients not only were more likely to used strong opioids than their counterparts without nonunion, but also used them for almost twice as long as those without nonunion (5.4 vs. 2.8 months, P < 0.001). Interestingly, although a higher proportion of nonunion patients used selective serotonin reuptake inhibitors (SSRI’s) than those without nonunion, the duration of SSRI therapy was shorter in the nonunion patients than in those without nonunion (8.1 vs. 12.1 months, P = 0.003).

**Figure 2:**
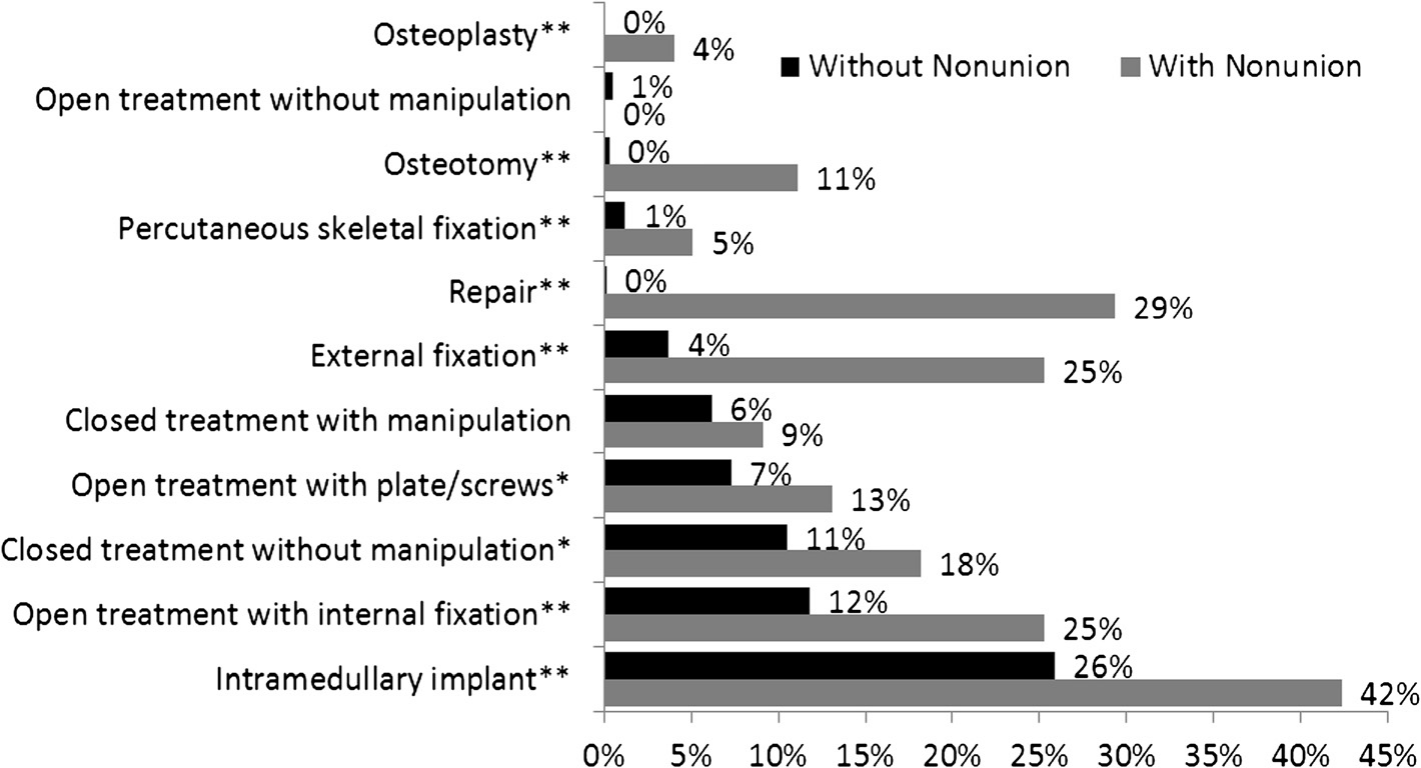
Post-fracture surgical procedures by nonunion status.

Nonunion patients received significantly more surgical procedures than their counterparts without nonunion (Figure 2). Almost a third (29%) of nonunion patients required repair, one in nine (11%) required osteotomy, and 4% required osteoplasty, whereas none of those without nonunion required these procedures. Almost five-fold difference existed between the rate of external fixation (25% vs. 4%, P < 0.0001) and percutaneous skeletal fixation (5% vs. 1%, P = 0.0045) in those with and without nonunion. Also, nonunion patients were more likely to receive an open treatment with internal fixation (25% vs. 12%, P = 0.0002) or plates and screws (13% vs. 7%, P = 0.044), a closed treatment without manipulation (18% vs. 11%, P = 0.023), or intramedullary implant (42% vs. 26%, P = 0.0005) than patients without nonunion. No statistically significant difference existed between patients with and without nonunion in two procedures: closed treatment with manipulation and open treatment without manipulation.

Other healthcare resources followed a similar pattern (Table 3). Nonunion patients were more likely to be hospitalized for reasons related to the tibia fracture (64.6% vs. 40.3%, P < 0.001) and other reasons (62.6% vs. 31.6%, P < 0.001) and to use tibia-related physical therapy (59.6% vs. 42.4%, P = 0.001) than their counterparts without nonunion. Other services were comparable between the two groups.

**Table 3:** Healthcare resource utilization in tibia shaft fracture patients by nonunion status

	**All tibia fractures N = 853: N (%)**	**With nonunion N = 99: N (%)**	**Without nonunion N = 754: N (%)**	**P Values †**
**Tibia Fracture Related Visits**				
Inpatient	368 (43.1%)	64 (64.60%)	304 (40.30%)	< 0.001
Outpatient	751 (88.0%)	86 (86.90%)	665 (88.20%)	0.7
Physical Therapy	379 (44.4%)	59 (59.60%)	320 (42.40%)	0.0012
Emergency Room	67 (7.90%)	6 (6.10%)	61 (8.10%)	0.48
Total	853 (100.00%)	99 (100.00%)	754 (100.00%)	NA
**Non-Tibia Fracture Related Visits**				
Inpatient	300 (35.20%)	62 (62.60%)	238 (31.60%)	< 0.001
Outpatient	853 (100.00%)	99 (100.00%)	754 (100.00%)	NA
Emergency Room	287 (33.60%)	41 (41.40%)	246 (32.60%)	0.082
Total	853 (100.00%)	99 (100.00%)	754 (100.00%)	NA
**Total Visits**				
Inpatient	529 (62.00%)	85 (85.90%)	444 (58.90%)	< 0.001
Outpatient	853 (100.00%)	99 (100.00%)	75 (400.00%)	NA
Emergency Room	324 (38.00%)	44 (44.40%)	280 (7.10%)	0.16
Overall Total	853 (100.00%)	99 (100.00%)	754 (100.00%)	

Notes: † P-value is based on the *T*-test.

### Medical costs

Medical costs of nonunion tibia shaft fracture patients were higher than of their counterparts without nonunion. This was true for services directly related to tibia fractures and other services, even if some of them had a comparable rate of use between the groups (Table 4 lists median and mean treatment costs with standard deviations). This was true for almost all types of healthcare services: directly related to tibia fracture and other services. This resulted in median total care cost for nonunion patients almost doubling that of patients without a nonunion ($25,555.97 vs. $11,686.24, P < 0.001). Median costs of inpatient services not related to tibia fractures were almost 20 times higher in nonunion patients than in those without nonunion ($7,214.43 vs. $368.81, P < 0.001). Although both groups used outpatient services with similar likelihood (Table 3), the median costs of these services were higher in the nonunion patients than in those without nonunion for both tibia related ($674.05 vs. $150.1, P < 0.001) and non-tibia related ones ($9,952.65 vs. $3,850.53, P < 0.001).

**Table 4:** Medical costs associated with tibia shaft fracture patients by nonunion status

**Medical and Pharmacy Costs**	**All Tibia Fractures N = 853**	**Nonunion N = 99:**	**Without Nonunion N = 754:**	**P values**†
**Tibia Fracture Related Costs**				
Inpatient				< 0.001
· Median	$0.00	$1,115.92	$0.00	
· Mean	$3,378.69	$7,263.96	$2,868.56	
· SD	$10,652.67	$16,129.49	$9,607.02	
Outpatient				< 0.001
· Median	$178.06	$674.05	$150.10	
· Mean	$584.24	$1,300.95	$490.14	
· SD	$1,505.94	$2,414.03	$1,315.75	
Emergency Room				0.52
· Median	$0.00	$0.00	$0.00	
· Mean	$32.34	$23.03	$33.56	
· SD	$261.46	$134.00	$273.86	
Total				< 0.001
· Median	$663.50	$2,316.07	$531.97	
· Mean	$3,995.27	$8,587.94	$3,392.25	
· SD	$10,811.50	$16,306.36	$9,721.82	
**Non-Tibia Fracture Related Costs**				
Inpatient				< 0.001
· Median	$611.98	$7,214.43	$368.81	
· Mean	$8,735.48	$25,487.19	$6,535.98	
· SD	$23,600.99	$51,161.42	$15,743.06	
Outpatient				< 0.001
· Median	$4,317.24	$9,952.65	$3,850.53	
· Mean	$8,836.56	$15,736.12	$7,930.64	
· SD	$13,962.48	$17,527.29	$13,172.68	
Emergency Room				0.049
· Median	$0.00	$0.00	$0.00	
· Mean	$260.65	$292.16	$256.52	
· SD	$1,314.07	$715.34	$1,373.70	
Total				< 0.001
· Median	$7,114.27	$18,630.94	$6,086.31	
· Mean	$17,832.68	$41,515.47	$14,723.14	
· SD	$31,298.95	$59,303.29	$23,817.82	
**Total Costs**				
Inpatient				< 0.001
· Median	$3,899.27	$9,697.55	$2,972.25	
· Mean	$12,114.17	$32,751.14	$9,404.54	
· SD	$26,874.18	$56,070.29	$18,565.20	
Outpatient				< 0.001
· Median	$4,869.33	$10,735.37	$4,419.38	
· Mean	$9,420.80	$17,037.08	$8,420.78	
· SD	$14,151.75	$18,219.82	$13,220.11	
Emergency Room				0.11
· Median	$0.00	$0.00	$0.00	
· Mean	$292.99	$315.19	$290.07	
· SD	$1,340.86	$732.55	$1,401.56	
Pain Medications				< 0.001
· Median	$110.12	$380.98	$92.75	
· Mean	$655.29	$1,034.90	$605.44	
· SD	$2,144.27	$1,633.62	$2,198.56	
Other Medications				0.61
· Median	$1,117.06	$1,117.47	$1,109.45	
· Mean	$2,274.94	$2,368.05	$2,262.71	
· SD	$3,539.38	$3,375.77	$3,562.27	
**Overall Total**				< 0.001
· Median	$13,364.57	$25,555.97	$11,686.24	
· Mean	$24,758.18	$53,506.36	$20,983.55	
· SD	$35,245.19	$65,259.77	$26,987.93	

Notes: † P-value is based on the Wilcoxon test. SD – standard deviation.

## Discussion

Tibia shaft fractures present a substantial burden on patients and the healthcare system. These fractures are common and cause physical limitations and pain to the patients. Nonunions further worsen the burden of tibia shaft fractures and lead to additional healthcare interventions [9,18]. Although nonunion can occur in any fracture site, tibia shaft is the most common among the long bones [19-21]. The extent of the healthcare burden and costs associated with tibia shaft fractures needs to be well understood to enhance the framework of decision making regarding treatment patterns and health insurance coverage, and to better evaluate the outcomes of nonunion fracture prevention and treatment. This study addressed an important gap in the literature: the lack of recent and broadly representative data on healthcare use and cost of tibia fracture treatment depending on the nonunion status.

Women with tibia fractures were found to be older than men, and this difference was statistically significant. Men tend to break their tibias in sport activities (e.g. soccer and skiing) or motor vehicle accidents [6]. In women, low bone mass and osteoporosis (common for old ages) are contributing factors to tibia fractures [22].

Pain control and medication use is an important issue in tibia shaft fracture treatment, especially when a nonunion is present [21]. Patients reported lower limb fractures as the most painful ones among fractures and injuries [23], which naturally drives the use of analgesics. Consistent with previous literature, we found opioids and NSAIDs to be commonly used for pain treatments among patients with bone shaft trauma [24-26]. Notably, other studies have shown a significant association between the use of NSAIDs [24,26] or opioids [26] with nonunion of long bone shaft fractures. We also found that patients with nonunion were more likely to experience additional fractures in the study period than those without nonunion, which could have increased their likelihood of receiving NSAIDs, opioids, or both. Although our study design precluded us from any inferences of causality, our results suggest that pain medication (especially opioid) use remains high in tibia shaft fracture patients with nonunion and may be a marker for nonunions.

Strong opioid use itself is a difficult issue in pain management of tibia fractures. Opioids are controlled substances that are associated with side effects, physical tolerance, withdrawal, and addiction [27]. We found substantially higher use of strong opioids in tibia fracture patients with nonunion than in those without nonunion. Because extended use of strong opioids may result in patient addiction, and drug misuse and diversion [28], previous research suggested cautious use of strong opioids [28]. Thus, prevention or prompt treatment of nonunions may not only help reduce pharmacy and medical costs, but may also lead to better control over opioid use.

Healthcare use and costs were higher in the nonunion group than in those without nonunion. Although the nonunion patients represented only 12% of the tibia shaft fracture patient population, their total costs were more than twice as much as those without a nonunion. This happened because nonunion patients were more likely to use all types of healthcare services than those without nonunion, and their per-patient cost of care was higher than in those without nonunion. We also found that nonunion patients underwent more surgical procedures post-fracture than their counterparts without nonunion. These findings are consistent with earlier reports suggesting that nonunion patients were more likely to be hospitalized [16,17] and operated on [15] than patients without nonunion. We found that both tibia and non-tibia related costs were higher in patients with a nonunion. This may indicate that tibia nonunion has an overall negative impact on other aspects of people’s health and causes further non-tibia-related healthcare resource use. Overall, our findings provide an update on medical costs of tibia shaft nonunion to the existing literature [15,18] and data on healthcare use in a broader patient population group than previously reported (BMP-7 treatment patients) [16,17].

While the healthcare system is under increasing financial burden, many healthcare institutions implement cost control strategies. Screening tibia shaft fracture patients for nonunion risks and addressing nonunions in a timely manner, may not only free up scarce healthcare resources and save healthcare dollars, but also improve patient outcomes [12]. The literature suggests that open fractures are more likely to result in a nonunion than closed ones [21,25,29,30]. Consistently, our study found that tibia shaft fracture nonunions were more likely to occur in those whose tibia fracture was open than in those with closed tibia fractures. Open status of a tibia shaft fracture may serve as a pre-cursor of nonunion.

The findings of this research should be interpreted in the context of the limitations of the study design. First, our study focused exclusively on patients with medical and prescription benefit coverage. Therefore, it may not be appropriate to draw inferences about broader populations (especially those without health care insurance) based on the findings presented here. Second, this study was descriptive in nature and did not investigate the effect of fracture-related characteristics (e.g., displaced, compound, or comminuted) or patient-related characteristics (e.g., smoking [31-33]) on the risk of nonunion. Smoking status, among other patient-related characteristics, was not available in the studied dataset. Third, the limitations of the databases precluded us from detailing specific within-hospital resource use (e.g., surgical theatre vs. other resources) or estimate whether all tibia fracture healed by the end of the study period.

Finally, we were unable to assess the burden of tibia shaft fracture nonunion beyond medical costs: e.g., on patients’ health-related quality of life or indirect costs to the society. Previous U.S. and the U.K. studies have found that the indirect costs of tibia fracture management are at least twice that of the direct healthcare costs [18,34,35]. Given the substantial burden of tibia shaft fractures not only on patients, but also on their family members and friends, there is a need to estimate indirect (e.g., productivity) costs of tibia shaft fractures and their nonunion.

## Conclusions

In conclusion, this retrospective descriptive analysis of patients with tibia shaft fractures revealed important differences between those with nonunion and their counterparts without a nonunion. Although we identified higher patient comorbidities, resource use, and costs associated with nonunions, many unanswered questions, especially as related to the indirect burden on society and risk factors for nonunion, still remain. Future research (preferably, through a prospective cohort or a placebo-controlled study) should investigate functional and productivity outcomes of tibia shaft fractures, risk factors for their nonunion, and the correlation between risk factors, such as opioid use, and additional fractures.

## Endnotes

^a^Carve-out services are medical services that are separated from a contract and paid under a different arrangement.

## Competing interests

All authors were employed by Eli Lilly and Company when the study was designed, analyses performed, results analyzed, and manuscript drafted. The authors declare no other competing interests.

## Authors’ contributions

All authors have made substantial contributions to conception and design of the study, or acquisition of data, or analysis and interpretation of data, have been involved in drafting the manuscript or revising it critically for important intellectual content and have given final approval of the version to be published. EA led the research concept and design, interpretation of the data, and was the primary writer of the manuscript. TKL contributed to the research design, lead data analyses, and contributed to manuscript writing. RB and JM contributed to research design, result interpretation, and manuscript writing. All authors read and approved the final manuscript.

## Pre-publication history

The pre-publication history for this paper can be accessed here:

http://www.biomedcentral.com/1471-2474/14/42/prepub

## Supplementary Material

Additional file 1 Index Tibia Fracture Diagnosis. Click here for file

## References

[B1] Praemer A Furner S Rice DP Musculoskeletal conditions in the United States 1992 Park Ridge, IL: American Academy of Orthopedic Surgeons

[B2] Miller NC Askew AE Tibia fractures. an overview of evaluation and treatment Orthop Nurs 2007 26 4 216 223 quiz 224–5 10.1097/01.NOR.0000284648.52968.27 17882096

[B3] AHRQ (Agency for Health Care Research and Quality) Introduction to the HCUP state inpatient databases (SID). Online Manual URL: http://hcup-us.ahrq.gov/db/state/siddist/Introduction_to_SID.pdf, Accessed 4 August 2011

[B4] Becker DJ Yun H Kilgore ML Health services utilization after fractures: evidence from Medicare J Gerontol A Biol Sci Med Sci 2010 65 9 1012 1020 20530242 10.1093/gerona/glq093

[B5] Bhandari M Guyatt GH Swiontkowski MF Schemitsch EH Treatment of open fractures of the shaft of the tibia J Bone Joint Surg Br 2001 83 1 62 68 10.1302/0301-620X.83B1.10986 11245540

[B6] Norvell JG Steele M Cooper TM Fracture, tibia and fibulahttp://emedicine.medscape.com/article/826304-overview. Updated 2011. Accessed April 4, 2011

[B7] Johnson B Christie J Open tibia shaft fractures: a review of the literature The Internet Journal of Orthopedic Surgery 2008 9 1 10.5580/1f16

[B8] Minoo P McCarthy JJ Herzenberg J Tibial nonunionshttp://emedicine.medscape.com/article/1252306-overview. Updated 2009. Accessed 4 April 2011

[B9] Alt V Donell ST Chhabra A Bentley A Eicher A Schnettler R A health economic analysis of the use of rhBMP-2 in gustilo-anderson grade III open tibial fractures for the UK, germany, and france Injury 2009 40 12 1269 1275 10.1016/j.injury.2009.02.007 19539926

[B10] Hak DJ Saleh K Socioeconomic burden of traumatic tibial fractures: nonunion or delayed union. Mescape 2001 URL: http://hcup-us.ahrq.gov/db/state/siddist/Introduction_to_SID.pdf, assessed 3 July 2011

[B11] Bhandari M Guyatt G Tornetta P 3rd Randomized trial of reamed and unreamed intramedullary nailing of tibial shaft fractures J Bone Joint Surg Am 2008 90 12 2567 2578 19047701 10.2106/JBJS.G.01694 PMC2663330

[B12] Kasturi G Adler RA Mechanical means to improve bone strength: ultrasound and vibration Curr Rheumatol Rep 2011 13 3 251 256 10.1007/s11926-011-0177-7 21484337

[B13] Martinez De Albornoz P Khanna A Longo UG Forriol F Maffulli N The evidence of low-intensity pulsed ultrasound for in vitro, animal and human fracture healing Br Med Bull 2011 100 39 57 10.1093/bmb/ldr006 21429948

[B14] Nolte PA van der Krans A Patka P Janssen IM Ryaby JP Albers GH Low-intensity pulsed ultrasound in the treatment of nonunions J Trauma 2001 51 4 693 702 discussion 702–3 10.1097/00005373-200110000-00012 11586161

[B15] Beaver R Brinker MR Barrack RL An analysis of the actual cost of tibial nonunions J La State Med Soc 1997 149 6 200 206 9188244

[B16] Dahabreh Z Calori GM Kanakaris NK Nikolaou VS Giannoudis PV A cost analysis of treatment of tibial fracture nonunion by bone grafting or bone morphogenetic protein-7 Int Orthop 2009 33 5 1407 1414 10.1007/s00264-008-0709-6 19052743 PMC2899110

[B17] Dahabreh Z Dimitriou R Giannoudis PV Health economics: A cost analysis of treatment of persistent fracture non-unions using bone morphogenetic protein-7 Injury 2007 38 3 371 377 10.1016/j.injury.2006.08.055 17070526

[B18] Heckman JD Sarasohn-Kahn J The economics of treating tibia fractures. the cost of delayed unions Bull Hosp Jt Dis 1997 56 1 63 72 9063607

[B19] Audige L Griffin D Bhandari M Kellam J Ruedi TP Path analysis of factors for delayed healing and nonunion in 416 operatively treated tibial shaft fractures Clin Orthop Relat Res 2005 438 221 232 16131895 10.1097/01.blo.0000163836.66906.74

[B20] Axelrad TW Kakar S Einhorn TA New technologies for the enhancement of skeletal repair Injury 2007 38 Suppl 1 S49 62 17383486 10.1016/j.injury.2007.02.010

[B21] Sakellarides HT Freeman PA Grant BD Delayed union and non-union of tibial-shaft fractures. a review of 100 cases J Bone Joint Surg Am 1964 46 557 569 14131432

[B22] Warriner AH Patkar NM Curtis JR Which fractures are most attributable to osteoporosis? J Clin Epidemiol 2011 64 1 46 53 10.1016/j.jclinepi.2010.07.007 21130353 PMC5030717

[B23] Kane RL Bershadsky B Rockwood T Saleh K Islam NC Visual analog scale pain reporting was standardized J Clin Epidemiol 2005 58 6 618 623 10.1016/j.jclinepi.2004.11.017 15878476

[B24] Giannoudis PV MacDonald DA Matthews SJ Smith RM Furlong AJ De Boer P Nonunion of the femoral diaphysis. the influence of reaming and non-steroidal anti-inflammatory drugs J Bone Joint Surg Br 2000 82 5 655 658 10.1302/0301-620X.82B5.9899 10963160

[B25] Bhandari M Tornetta P 3rd Sprague S Predictors of reoperation following operative management of fractures of the tibial shaft J Orthop Trauma 2003 17 5 353 361 10.1097/00005131-200305000-00006 12759640

[B26] Bhattacharyya T Levin R Vrahas MS Solomon DH Nonsteroidal antiinflammatory drugs and nonunion of humeral shaft fractures Arthritis Rheum 2005 53 3 364 367 10.1002/art.21170 15934108

[B27] Dellemijn PL Opioids in non-cancer pain: A life-time sentence? Eur J Pain 2001 5 3 333 339 10.1053/eujp.2001.0240 11558990

[B28] Brushwood DB Rich BA Coleman JJ Bolen J Wong W Legal liability perspectives on abuse-deterrent opioids in the treatment of chronic pain J Pain Palliat Care Pharmacother 2010 24 4 333 348 10.3109/15360288.2010.524979 21133741

[B29] Karladani AH Granhed H Karrholm J Styf J The influence of fracture etiology and type on fracture healing: A review of 104 consecutive tibial shaft fractures Arch Orthop Trauma Surg 2001 121 6 325 328 10.1007/s004020000252 11482464

[B30] Boyd HB Lipinski SW Wiley JH Observations on non-union of the shafts of the long bones, with a statistical analysis of 842 patients J Bone Joint Surg Am 1961 43 159 168

[B31] Castillo RC Bosse MJ MacKenzie EJ Patterson BM LEAP Study Group Impact of smoking on fracture healing and risk of complications in limb-threatening open tibia fractures J Orthop Trauma 2005 19 3 151 157 10.1097/00005131-200503000-00001 15758667

[B32] Harvey EJ Agel J Selznick HS Chapman JR Henley MB Deleterious effect of smoking on healing of open tibia-shaft fractures Am J Orthop (Belle Mead NJ) 2002 31 9 518 521 12650537

[B33] Schmitz MA Finnegan M Natarajan R Champine J A comparison of the relative costs of cast treatment and intramedullary nailing for tibial diaphyseal fractures in the UK Injury 1997 28 5–6 373 375 9764236 10.1016/s0020-1383(97)00028-4

[B34] Downing ND Griffin DR Davis TR A comparison of the relative costs of cast treatment and intramedullary nailing for tibial diaphyseal fractures in the UK Injury 1997 28 373 375 10.1016/S0020-1383(97)00028-4 9764236

[B35] MacKenzie EJ Morris JA Jr Jurkovich GJ Return to work following injury: The role of economic, social, and job-related factors Am J Public Health 1998 88 11 1630 1637 10.2105/AJPH.88.11.1630 9807528 PMC1508559

