# Supplementary material for: Tibia shaft fractures: costly burden of nonunions
Source: BMC Musculoskelet Disord. 2013 Jan 26;14:42. doi: 10.1186/1471-2474-14-42 (PMC3573940; doi:10.1186/1471-2474-14-42)
Supplement: Additional file 1 — Index Tibia Fracture Diagnosis. [file 1471-2474-14-42-S1.doc]

| **1/01/2005**  **12/31/2008**  **Index Tibia Fracture Diagnosis**  **Index Date**  **1/01/06-12/31/06**  **Index Period**  **12 Months Before Index Date 24 Months After Index Date**  **Prior Period Post/Follow-up Period** |
| --- |

Study timeline
